# Fibroblast heterogeneity and functions: insights from single-cell sequencing in wound healing, breast cancer, ovarian cancer and melanoma

**DOI:** 10.3389/fgene.2024.1304853

**Published:** 2024-03-08

**Authors:** Omar Lujano Olazaba, Jeffrey Farrow, Teresa Monkkonen

**Affiliations:** Department of Biology, San Diego State University, San Diego, CA, United States

**Keywords:** wound healing, cancer associated fibroblast, single-cell multiomics, breast cancer, ovarian cancer, melanoma

## Abstract

Cancer has been described as the wound that does not heal, in large part due to fibroblast involvement. Activation of cancer-associated fibroblasts (CAFs) contributes to critical features of the tumor microenvironment, including upregulation of key marker proteins, recruitment of immune cells, and deposition of extracellular matrix (ECM)—similar to fibroblast activation in injury-induced wound healing. Prior to the widespread availability of single-cell RNA sequencing (scRNA seq), studies of CAFs or fibroblasts in wound healing largely relied on models guided by individual fibroblast markers, or methods with less resolution to unravel the heterogeneous nature of CAFs and wound healing fibroblasts (especially regarding scarring outcome). Here, insights from the enhanced resolution provided by scRNA sequencing of fibroblasts in normal wound healing, breast cancer, ovarian cancer, and melanoma are discussed. These data have revealed differences in expression of established canonical activation marker genes, epigenetic modifications, fibroblast lineages, new gene and proteins of clinical interest for further experimentation, and novel signaling interactions with other cell types that include spatial information.

## 1 Introduction

Fibroblasts originate from primary mesenchyme cells that contribute to the formation of the mesoderm during embryonic development (Kalluri and Weinberg, 2009). This embryonic origin is shared by other mesenchymal lineages, including adipocytes, chondrocytes and osteoblasts (Sahai et al., 2020). In adults, fibroblasts are identified by their spindle-shaped morphology in the interstitial stroma connecting the tissue parenchyma (Tarin and Croft, 1969; Majno et al., 1971). Generally, it is thought that fibroblasts are quiescent but poised for activation as needed or induced. Fibroblast activation is initiated by the surrounding stroma with disrupted tissue homeostasis, diseases inducing fibrosis, or cancer (Monaco and Lawrence, 2003; Gurtner et al., 2008; Kalluri, 2016). Activation induces fibroblast proliferation, migration, ECM remodeling, and paracrine interactions with other cells including endothelial or immune cells (Kalluri, 2016). Thus, fibroblasts could be considered master regulators of tissue homeostasis, biomechanical forces, and structure--and these functions can be hijacked in disease (Tomasek et al., 2002).

Some critical proteins expressed by activated fibroblasts often include, but are not limited to, alpha Smooth Muscle Actin (aSMA), Fibroblast Activating Protein (FAP), Vimentin (VIM), TGFb, and Fibroblast Specific Protein-1 (FSP1/S100A4) (Tomasek et al., 2002; Roberts et al., 2013). Fibroblast activation is reversible in normal wound healing, while chronic activation of fibroblasts in cancer is less reversible, in part due to epigenetic alterations (Zeisberg and Kalluri, 2013; Zeisberg and Zeisberg, 2013; Tampe and Zeisberg, 2014). Persistent fibroblast activation is associated with pathologies including cancer and lung and kidney fibrosis (Selman and Pardo, 2002; Tampe and Zeisberg, 2014; Kalluri, 2016).

Other hallmark features and challenges in studying fibroblasts are their well-established plasticity and diverse lineages. Fibroblasts arise from diverse cell types including bone marrow derived-mesenchymal stem cells, adipose-derived precursors, tissue-resident fibroblasts, pericytes, endothelial cells, and mesothelial cells, as reviewed elsewhere (Bojin et al., 2012; Micallef et al., 2012; Kalluri, 2016; LeBleu and Kalluri, 2018). The various origins of activated fibroblasts reflects the heterogeneity and diverse functions of fibroblasts, and fits with the lack of a single marker identifying all activated fibroblasts either in cancer or wound healing.

Prior to widespread use of scRNA seq data unraveled fibroblast origin, heterogeneity, and function by functional analysis of canonical activation markers, transgenic mice, co-culture studies, and bulk RNA sequencing. While these approaches made remarkable advances, single cell RNA sequencing (scRNA seq) and spatial transcriptomics have improved synchronous investigation of fibroblast heterogeneity and intracellular interactions in depth. This review addresses scRNA sequencing data from wound healing, breast cancer, ovarian cancer, and melanoma cancer associated fibroblasts (CAFs) - highlighting key insights, new understanding of intercellular interactions, and gaps in knowledge. We highlight data from breast and ovarian cancer as two solid tumor women’s carcinomas where CAFs play a relatively similar established role, and melanoma to compare and contrast with normal wound healing. Other reviews have addressed CAF heterogeneity broadly across many cancers, and generated a general framework of fibroblast subpopulations (Kalluri, 2016; Sahai et al., 2020; Hasan and Buechler, 2022; Lavie et al., 2022; Luo et al., 2022).

## 2 Single-cell sequencing of fibroblasts in wound healing

Wound healing is a process where fibroblasts activate, proliferate, and deposit extracellular matrix (ECM). Previously, wound healing fibroblasts were categorized as papillary fibroblasts associated with regeneration, reticular fibroblasts which promote scarring (Sorrell et al., 2004; Janson et al., 2012), and a hair follicle adjacent fibroblast population. Papillary and reticular fibroblasts are functionally distinct regarding growth factor and ECM secretion, but lacked established marker proteins as of 2012 (Janson et al., 2012). Wound healing fibroblasts can also arise from mesenchymal stem cells, fibrocytes, or endothelial cells, and much intracellular signaling had been established prior to widespread use of scRNA seq (Darby et al., 2014). Scarring ability of fibroblasts is associated with loss of expression of Engrailed-1 (*En1*) and are termed engrailed past fibroblasts (EPFs) (Jiang et al., 2020). Current wound healing research is focused on dissecting the regulation of regenerative wound healing-a favorable result characterized by hair neogenesis; decreased contraction; decreased WNT and TGF-B signaling; and decreased collagen production-versus fibrotic wounds with decreased hair neogenesis, increased contraction, increased WNT and TGF-B signaling, and increased collagen production (Joost et al., 2018). Elucidating the molecular differences between fibrotic and regenerative wound healing has implications for many diseases (Chen et al., 2022).

### 2.1 SCRNA sequencing and multi-omics analysis of wound healing fibroblasts in mouse models

Wound healing experiments have models with established timepoints for evaluating wound healing, which together with lineage tracing and transgenic mice, creates opportunities for targeted spatial, multimodal analysis. A recent study characterized wound healing fibroblast heterogeneity using lineage tracing, spatial transcriptomics, scRNA-seq and scATAC-seq (Foster et al., 2021). Imaging over time in mice including intravital imaging showed that different resident fibroblast populations activate, differentiate, and migrate inward (Foster et al., 2021). Expression of the established wound healing fibroblast markers *Pdgfr-a*, *En-1*, and *Cd26* was variable throughout wound tissue by spatial transcriptomics, suggesting functional heterogeneity among the fibroblasts activated by injury (Foster et al., 2021). In terms of canonical markers, *Pdgfra* was elevated in the inner and outer wound, while alpha smooth muscle actin and fibroblast activating protein were not (Foster et al., 2021). The local mechanofibrotic fibroblasts are recruited to the wound bed immediately after wounding, and proliferate (Foster et al., 2021). During re-epithelialization, mechano-fibrotic fibroblasts differentiate, migrate to the wound center, and mature into *Spp1+* profibrotic proliferator fibroblasts with overall increased chromatin accessibility. Simultaneously, *Fn1* remodeling fibroblasts appear in the outer deep dermis with *Thbs1* expressing fibroblasts expressing chemokines, resulting in wound closure.

### 2.2 scRNA sequencing and wound healing fibroblast heterogeneity

**FIGURE 1:**
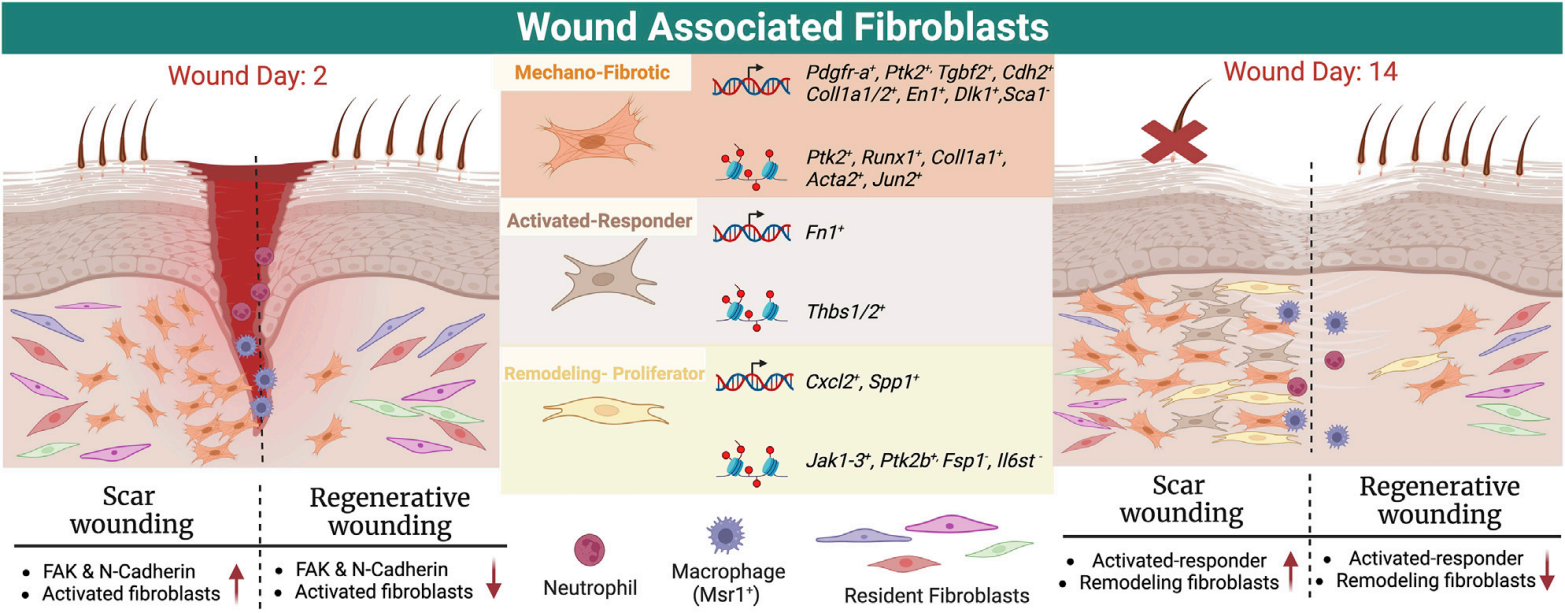
Schematic showing subtypes of wound healing fibroblasts and their location in early and late/completed wound healing, together with markers from scRNA sequencing data and ATAC seq.

The mechano-fibrotic cluster showed high *Col1a1, Acta2*, and *Pdgfra* accessibility, as well as Focal Adhesion Kinase (*Fak*) and downstream elements such as *Jun*, consistent with mechanoresponsive and fibrotic roles (See Table 1). Cluster-wide enrichment analysis revealed enrichment for fibroblast migration, focal adhesion and FAK-pathway signaling response elements in the mechanofibrotic cells (Foster et al., 2021). Temporal analysis of scRNA-ATAC data showed epigenetic progression associated with differentiation into the other three populations was driven by mechanical signaling. Subsequent *in vivo* experiments showed that FAK inhibited wounds healed at the same rate, but displayed smaller and thinner scars, less dense ECM, downregulation of mechanotransduction and fibrotic pathways, and fewer mechanofibrotic fibroblasts at completion of wound healing (Foster et al., 2021). FAK inhibition also elicited smaller, disordered clones, showing FAK importance in differentiation (Foster et al., 2021). Mechanofibrotic populations were most abundant at 7 days post wound, and decreased by completion of wound healing (Foster et al., 2021). See Figure 1 for a summary of normal wound healing.

**TABLE 1:** Wound healing fibroblast populations, ATAC, and scRNA seq data of genes associated with mechano-fibrotic, activated-responder, and remodeling-proliferator fibroblasts in normal wound healing.

Functional classification	Single-cell ATAC-seq markers	Single-cell RNA-Seq markers	References
Mechano-Fibrotic	*Col1a1, Acta2, Pdgfra, Ptk2*	*Pdgfr-a, Ptk2, Tgfb2, Chd2, Coll1a1, Coll1a2, Mest, Plac8, Col3a1, Col5a1 Timp1, Timp2, Mmp3*	Foster et al. (2021), Phan et al. (2021), Phan et al. (2020)
Activated-Responder	*Thbs1, Thbs2*	*Fn1, Thbs1*	Foster et al. (2021)
Remodeling-Proliferator	*Jak1, Jak2, Jak3, Ptk2b*	*Cxcl2, Spp1, Crabp1*	Foster et al., 2021, Phan et al. (2021)

These results align with other reports of mechanical properties driving scar formation; expression of FAK (Foster et al., 2021), N-cadherin (Jiang et al., 2020), or other mechano-related proteins can result in deep scars (Wong et al., 2011). EPF lineage tracing and limited intravital microscopy in transgenic mice showed EPFs aggregating in the deep wound, migrating to the wound center, then migrating toward the epidermis requiring N-Cadherin-mediated cell-cell adhesion (Jiang et al., 2020). Human skin fascia explants from 71 donors, or cultured scar-like tissues derived from mice and human samples showed that fascia-derived EPFs are the major contributor of scar, and largely express a-SMA (76%) and DLK-1 (52%) similar to mouse models at 7 days post wound, as confirmed by immunostaining (Jiang et al., 2020). The EPF cells collectively migrate in swarms toward the wound center. Another scRNA seq study in mouse models showed a *Mest+ Plac8+* lower wound fibroblast population correlating with scar formation (Phan et al., 2021).

The remodeling and proliferator fibroblast clusters are associated with regenerative wound healing and arise from the less differentiated mechanofibrotic fibroblasts according to ATAC-seq and Visium analysis (Foster et al., 2021). Activated-responder fibroblasts display elevated *Fn1* and *Thbs1* expression, while the remodeling fibroblast cluster showed increased *Jak2* accessibility, with decreased *Fsp1* and *Il6st* accessibility. The proliferator cluster displayed increased *Ptk2b*, *Jak1*, and *Jak3* accessibility (Foster et al., 2021). This study leveraged multiple unbiased methodologies together with lineage tracing and experimental follow-up, has many strengths and represents an excellent framework for characterizing fibroblast heterogeneity.

Another study comparing regenerating, scarring, homeostatic, and developing skin in mice using scRNA seq identified *Lef* positivity in neonatal papillary fibroblasts, which represented a transient regenerative fibroblast population (Phan et al., 2020). ScRNA seq showed increased expression of collagens and ECM remodeling enzymes (*Col1a1, Col1a2, Col3a1, Col5a1 Timp1, Timp2, Mmp3*) as well as *Cxcl1, Cxcl10, Fn1,* and *S100A14* in scarring fibroblasts at day 21 (Phan et al., 2020). Velocity analysis suggests that the pathways contributing the most to fibroblast heterogeneity included integrin, Wnt, cadherin, and TGF-b. Subsequent mouse studies showed that *Lef1* positive fibroblasts, enriched in the papillary *Dpp4+* fibroblast clusters, enriched regenerative wound healing in adults in a wound which usually scars (Phan et al., 2020). This study offers an example of how scRNA seq and functional analysis of a marker gene can inform each other and lead to exciting *in vivo* data.

ScRNA seq analysis of closed murine wounds revealed 5 fibroblast populations (Guerrero-Juarez et al., 2019). Fibroblasts displayed a common 20 transcription factor signature including *Tcf4*, *Runx1*, and *Zeb2*, consistent with previous literature (Guerrero-Juarez et al., 2019). *Twist1/2*, *Sox11*, *Foxp1*, and *Id2/3* were transcription factors defining fibroblast subpopulations (Guerrero-Juarez et al., 2019). There was heterogeneity among some canonical activation markers, including *Tgfbr2/3* and *Pdgfra/b* within the fibroblast populations, suggesting the need to refine our understanding of these activation markers; for example, *Pdgfra* high fibroblasts included both lower and upper wound healing fibroblasts (Guerrero-Juarez et al., 2019). Pseudotime and RNA velocity analysis suggest the presence of two major populations, *Crabp1+,Pdgrfa* ^*hi*^ and *Pdgfr* ^*lo*^ fibroblasts, with further sub-populations of fibroblasts at different stages of differentiation. *Col1a1* and *Mest* were associated with early cell fates in these trajectories, consistent with mechanofibroblast data (Foster et al., 2021). These data also revealed gene signatures associated with hematopoietic lineages in a rare genetically identified contractile fibroblast population, which was supported *by in vivo* follow up in mice (Guerrero-Juarez et al., 2019). These data generate useful hypotheses regarding wound healing fibroblast differentiation, leveraging fresh murine tissues and velocity analysis. Further lineage tracing and co-localization using strategies such as RNA FISH will be useful to address these putative pathways.

Recent data on normal human dermal fibroblasts provides context for wound healing fibroblast data. SCRNA seq of normal human fibroblast samples revealed that papillary fibroblasts (pFIB) expressed high levels of previously identified markers *OL6A5*, *COL23A1* and *HSPB3* as expected, along with the Wnt inhibitors *APCDD1* and *WIF1, NTN1, and PDPN* (Agnes et al., 2023). The reticular fibroblast cluster (rFIB) displayed high *THY1*, *FMO1*, *MYOC, LSP1*, *MGP* and *ACTA2* expression in addition to the preadipocyte associated markers *PPARG* and *CD36*. The only established dermal fibroblast marker that did not behave as expected was *DPP4*; although associated with papillary fibroblasts in the literature, it was present in both pFIB and rFIB populations in this study (Agnes et al., 2023). C*D90/THY1*, often used as the sole identifier for skin fibroblasts, was only expressed by skin fibroblasts in this analysis (Agnes et al., 2023). Of note that the majority of CAFs expressed *CD90* in this study (Agnes et al., 2023). These gene expression profiles are useful context for wound healing fibroblast markers, for instance, highlighting that *ACTA2* and *PDPN* would not be appropriate.

### 2.3 Intercellular interactions of fibroblasts in wound healing

Comparison of fibrotic and regenerative murine wounds after the same initial large wound showed that *EN1-*negative myofibroblasts expressed *Aebp1*, *Col1a1/2*, *Col3a1*, *Col8a1*, *Eln*, *Mfap2*, *Mfap4*, and *Sparc*; while EN1-positive myofibroblasts expressed *Birc5*, *Pclaf*, *Stnm1*, *Ube2c*, *Hist1h2ap*, *Col5a3*, *Cks2*, *Aqp1*,*Tnfaip6*, and *Timp1* (Chen et al., 2022). Ligand-receptor analysis of myofibroblasts and macrophages revealed changes in signaling modulating morphogenesis, migration, motility, and TGFb in both macrophages and fibroblasts (Chen et al., 2022). Both fibroblasts and macrophages of fibrotic wounds display decreased hair neogenesis, and increased contraction, Wnt and TGF-b signaling, and collagen production. Fibrotic and regenerative myofibroblasts displayed differential gene expression associated with mRNA metabolism and organelle organization. These data support that fibrotic wounds had more contractile, EN1-positive fibroblasts which interact with macrophages to induce Wnt, Tgfb signaling, and collagen production (Chen et al., 2022). *In vitro* experiments suggested *En1* loss facilitated migration. Comparison of fibrotic and regenerative macrophages highlighted differences in innate and adaptive immunity signaling, antigen presentation and phagocytosis. In contrast to CAFs, pericytes could generate their own cluster here. This robust study adds to the understanding of fibroblast-macrophage signaling in regenerative versus fibrotic wound healing, using wounds inflicted the same way that have healed differently, together with *in vitro* follow up. These gene expression signatures, in particular if recapitulated in human studies, would be of clinical interest to reduce persistent inflammation and fibrosis.

ScRNA seq of regenerative or scarring murine wounds (based on depth, size, and age) suggested that a key characteristic of regenerative wounds is a higher proportion of upper wound fibroblasts (Phan et al., 2021). This analysis also identified a *Tgfbi* cluster, a distinct, novel wound healing fibroblast population displaying characteristics of lower wound fibroblasts. *Crabp1+* upper wound fibroblasts are proximal to the wound epithelium and are thought to be required for hair neogenesis in wounding (Phan et al., 2021). Velocity analysis revealed that in both scarring and regenerative wounds, upper wound fibroblasts closely associate with dermal papilla, suggesting upper wound fibroblasts are required for hair follicle regeneration, and that papillary fibroblasts may migrate from the wound periphery to the center of the wound (Phan et al., 2021). Velocity analysis suggested that overall, the wound healing fibroblasts in regenerative and scarring wounds are similar populations- but different in numbers. This work is another useful comparison of regenerative versus scarring wounds. The sequencing data, combined with sorting based of *Hic1+* reporter cells represents an excellent strategy to investigate novel wound healing fibroblast markers; this study revealed that *Hic1+* fibroblasts do not contribute to regenerative wounds and thus do not broadly identify dermal fibroblasts as previously hypothesized. One limitation is the initial clustering based on previous markers. We look forward to studies elucidating *Tgfbi* function in wound healing fibroblasts.

Other data from transgenic *Lef1* reporter mice shows that regenerative fibroblasts may require activation by epithelial stem cells. Labeling of stem/progenitors in the hair follicle showed that *Lgr5/6* rapidly induced a genetic wound signature that, for *Lgr5* progeny, included the remodeling *Itgb1, Cd44,* and *Thbs1* receptors which interact with the wound environment, as verified by FISH (Joost et al., 2018). This stem cell niche may be further supported by the lymphatic structures surrounding them (Ge et al., 2020). Data suggest that the stem cells work with papilla-derived upper wound fibroblasts in wound regeneration (Joost et al., 2018). Future studies may expand and verify functions of some of these stem cell-dependent gene signatures.

## 3 Single-cell sequencing of breast CAFs

Breast cancer is one of the cancers where CAFs have been most deeply scrutinized. Breast cancer is broadly classified into luminal, HER2, and triple-negative or basal-like subtypes with different biology and patient outcomes (Sørlie et al., 2003; Bastien et al., 2012). Numerous correlations have been reported between breast cancer CAFs and patient prognosis (Paulsson and Micke, 2014). In human breast cancer, high aSMA or PDGFRb+ myofibroblast infiltration was associated with poor patient prognosis, and high PDPN expression is associated with higher tumor grade (Toullec et al., 2010; Paulsson and Micke, 2014). FAP has associated with unaltered or potentially favorable prognosis (Ariga et al., 2001; Tchou et al., 2013), and the FSP data conflict as well (Park et al., 2016). TGFbR2 has also been associated with longer survival (Paulsson and Micke, 2014). These studies can rely heavily on antibodies lacking full validation, and can be qualitative/subjective. High stroma to tumor ratio in TNBC samples was associated with poorer prognosis (Moorman et al., 2012). It is important to note that these correlations do not address causality, interactions with other cells, or subtype-specific differences between the microenvironment (i.e., desmoplasia association with TNBC). Prior to scRNA sequencing, data showed that no single canonical CAF marker identified all CAFs, and reflected canonical marker heterogeneity (Mao et al., 2013). Of note, other breast cancer CAF markers include CAV1 and P53 (Mao et al., 2013).

### 3.1 SCRNA sequencing data from mouse models

Sequencing the M6 mouse TNBC model showed that CAF expression of *Col4a1, Tspan11, St8sia2,* and *Tnfaip6* was dependent on hedgehog signaling (Cazet et al., 2018). SMO inhibition inhibited expression of these genes, which were correlated with poor prognosis, while not altering CAF content (Cazet et al., 2018). Other scRNA sequence data of CAFs over *MMTV-PyMT* tumor progression revealing that late stage tumors with higher CAF content had worse survival (Elwakeel et al., 2019). Late-stage CAFs showed enrichment for *Rgs5,* Nfkb pathway, *Serpins* (a1d,a3f, e2, and i1), *Timp1*, *Acta2* expression, and vCAF signatures, while *Pdgfrb* decreased with tumor progression (Elwakeel et al., 2019). Other classifications of developmental and cycling CAFs were identified as well (Elwakeel et al., 2019).

SCRNA seq analysis of 4T1 TNBC tumors and Balb/c mouse mammary fat pad fibroblasts was done after excluding tumor and immune cells, revealing 6 CAF clusters (versus 3 clusters without these steps). These clusters consisted of *Acta2+* myCAFs, *Ly6c+* iCAFs, MHC class II apCAFs, *Cd53+* CAFs, *Cd74+* CAFs, and *Crabp1+* CAFs (Sebastian et al., 2020). The *Col1a1, Postn, Pdgfra,* and *Dcn* canonical CAF markers were widely expressed in normal mammary fibroblasts, while *Pdpn* and *Thy1* expression was only in *Ly6c+* and *Acta2+* CAF clusters. The *Pdgfra* canonical CAF marker was more highly expressed in *Ly6c+* iCAFs, while *Acta2+* myCAFs had higher *Pdgfrb* expression, and *Cd74+* CAFs displayed the highest *Fsp* expression. Here, normal fibroblasts had higher gene expression than CAFs. This work is commendable in re-doing scRNA seq to thoroughly capture CAF heterogeneity, and leveraging a mouse model to compare with normal (although this work relied solely on *Pdgrfa* to define normal fibroblasts). It would be interesting examine these signatures in other TNBC models and patient samples.

### 3.2 SCRNA sequencing CAF data from subtypes of breast cancer

CAF subpopulations from specific datasets associated with a breast cancer subtype fit with general CAF categorization schemes, and identify some novel genes. Analysis of 11 TNBCs correlated *SPARC* expression with high *POSTN* expression and worse survival (Alcaraz et al., 2023). Here, *SPARC* was expressed in both iCAFs and myCAFs (Alcaraz et al., 2023). Another analysis of TNBC and normal samples together with mass spectrometry showed that biglycan expression was high in tumors with high CAF infiltration versus normal fibroblasts (Zheng et al., 2022). High biglycan expression correlated with high expression of ECM, lymphangiogenesis, EMT, angiogenesis, and TGFb associated genes including *CD14*, *TGFB3*, *ENTPD1*, *NT5E* and *BMP1*; but negatively correlated with T cell infiltration (Zheng et al., 2022). BGN expression was highest overall in CAFs (Zheng et al., 2022).

A third TNBC focused analysis classified CAFs into myCAFs (*COL1A1/2, ACTA2, FAP, PDPN*) with *ZEB1* and *FOXP1* transcription factor enrichment; and iCAFs (*CXCL2, FGF7/10, BMP4/7, HGF*, and *IGF1*) (Wu et al., 2020) displaying high *EGFR*, *TCF4*, and stem cell transcription factor enrichment. Clustering also identified a new perivascular-like cell type associated with immune evasion (Wu et al., 2020). MyCAFs colocalized with invading tumor fronts while iCAFs did not, as observed by immunohistochemistry (Wu et al., 2020). Analysis of ligand/receptor interactions showed that iCAFs strongly interacted with myeloid cells via complement signaling, and a subset of iCAFs showed a signature correlating with T cell exhaustion (Wu et al., 2020). A scRNA sequencing analysis of luminal/HER2 breast cancer yielded two CAF populations: myofibroblasts with high *POSTN* and collagen, or *PLAG2G2A+* CAFs with high *OGN* associated with immune recruitment (Liu et al., 2022). *PLAG2G2A+* CAFs had more interaction with macrophages, dendritic cells, and T cells, verified by co-culture, as well as *APOD* expression. *APOD* was also enriched in luminal relative to HER2+ breast cancer (Liu et al., 2022).

Pal et al sequenced many patient and normal samples, identifying specific genes highly expressed in HER2, TNBC, BRCA and ER+ CAFs (Pal et al., 2021). Data from n = 8–13 patients/subtype show overlap in high *WISP2, SFRP2/4*, and *COL8A1* expression across a few subtypes, with many distinct genes as well, and identify a distinct pericyte+ CAF population in HER2+ cancers. This work also clustered of pre- and post-menopausal fibroblast populations from n = 5–8 women, showing 5 normal fibroblast clusters and overall fewer fibroblasts after menopause. This broad study is an excellent starting point or atlas for fibroblast comparisons within different patients and biological contexts, including normal fibroblasts as a reference and supporting microscopy, and gives rise to many interesting similarities and differences in gene signatures worth exploring. Highlights of these studies includes excellent experimental validation of ligand-receptor interaction findings and immunostaining for protein levels, and uncovering new stemness and EMT-related genes. Many of these studies focus on correlations with prognosis, which need further investigation.

### 3.3 SNRAN sequencing of human breast CAFs

Other studies have used scRNA sequencing to broadly investigate breast cancer broadly. In 5 luminal and 6 TNBC breast tumors, scRNA sequencing and immunostaining showed that high Fsp to Pdpn Fsp CAF ratio was associated with BRCA mutations, and that these genes separated the main CAF populations (Friedman et al., 2020). PDPN+ CAFs decreased over tumor progression, and were rarely present in metastases, and displayed longer cell morphology (Friedman et al., 2020). *Fsp+* CAFs included antigen presenting and protein folding/metabolic high enriched CAF subtypes (Friedman et al., 2020). Another analysis showed that IGKV3-11 CAF expression was correlated with poor survival, while ribosomal genes including *RPS9*, *RPS8*, *RPS6*, *RPL38*, and *RPL34* correlated with favorable outcome (Huang et al., 2023). A different study showed a 5 cytokine gene signature (*EDIL3, GRP, IL16, PTN,* and *TAC1*) in CAFs predicted for poor patient survival, higher tumor cell heterogeneity, and aggressive disease (Sun et al., 2021), while other analysis of 5 human breast tumors found high CAF FGFR levels was associated with CAF proliferation, and altered cytokine expression including VCAM1(Wu et al.). The FGFR data were supported by *in vitro* follow up experiments. A different comparison of human data showed that *PRRX1,* present only in breast cancer CAFs, correlated with canonical CAF marker expression and perpetually activated CAFs (Chung et al., 2021). Additional velocity analysis suggested that iCAFs give rise to myCAFs (Chung et al., 2021). These data correlate disease prognosis with selected gene signatures in CAFs, including ribosomal genes that predict favorable prognosis. However, to what extend these target genes will yield clinical application has yet to be determined.

### 3.4 Spatial transcriptomics of breast CAFs

**FIGURE 2:**
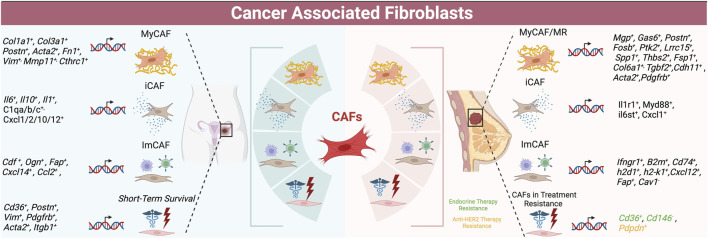
Schematic of CAF populations in ovarian (left) and breast cancer (right) -- summarizing subtypes with highest concordance as well as markers of treatment resistance and survival.

SCRNA seq and spatial transcriptomics of early and late *MMTV-PyMT* tumors showed CAFs clustering into six subgroups across the broader categories of steady state-like (SSL), mechanoresponsive (MR) and immunomodulatory (IM) fibroblasts (Foster et al., 2022). SSLCAFs express *Pi16* and *Dpp4,* with a high CD34 cluster and increased accessibility to *Dpp4*, *Ly6a*, and *Cd34* by ATACseq, and another cluster high in cytokine/and growth factor expression (*BMP1,3* and *Wnt2*). MRCAFs had elevated expression of mechanosensing mediators and ECM components, with one cluster displaying high focal adhesion kinase (FAK) associated expression (*Mgp, Gas6, Postn, and Fosb*) with high *Fos*, *Fosb*, *Junb*, *Jund*, and *Runx1/2* accessibility (see Table 2 and Figure 2). Another cluster had high *Lrrc15, Spp1,* and ECM gene expression with high *Gas6*, *Yap*, and *Acta2* accessibility. A MRCAF subpopulation had high expression of fibrosis-associated factors, *Thbs2*, *Fsp1*, *Col6a1*, and *Cdh11*. These subpopulations support the upregulation of focal adhesion, integrin binding, protein binding involved in cell-matrix adhesion, and PI3K- AKT-mTOR-signaling characteristic of MR fibroblasts. IMCAFs displayed high type II interferon and IL1 signaling (*Il1r1*, *Myd88*, *il6st*, and *Cxcl1*) and antigen presentation genes (*Ifngr1, B2m, Cd74, H2-D1, H2-K1,* and *Cxcl12*) with high *Cxcl12*, *Il6*, and *Ccl19* accessibility (Foster et al., 2022). The highest heterogeneity was present in early tumors, while late tumors had fewer CAFs equally distributed across the 8 clusters.

**TABLE 2:** Breast cancer CAF populations, ATAC, and scRNA seq data of genes associated with CAFs in breast cancer along with genes associated with treatment resistance.

Functional classification	Single-cell ATAC-seq markers	Single-cell RNA-Seq markers	References
MyCAF/MR	*Fos*, *Fosb*, *Junb*, *Jund*, *Runx1/2, Gas6*, *Yap*, *Acta2*	*Mgp, Gas6, Postn, Fosb, Ptk2, Lrrc15, Spp1, Thbs2, Fsp1, Col6a1, Tgbf2, Cdh11, Acta2, Pdgfrb,* *Dcn, Lum,Vcan, Col14a1, Fbln1, Fbln2, Smoc, Lox, Loxl1,Cxcl14*	Foster et al. (2022), Bartoschek et al. (2018)
iCAF	none reported	*Il1r1, Myd88, Il6st, Cxcl1 CXCL2, FGF7, FGF10 BMP4/7, HGF, IGF1*	Foster et al. (2022), Wu et al. (2020)
ImCAF	none reported	*Ifngr1, B2m, Cd74, H2-D1, H2-K1, Cxcl12*	Foster et al. (2022)
CAFs in Treatment Resistance	None reported	*PDPN, CD63, CD146*	Du et al. (2023), Gao et al. (2020), Brechbuhl et al. (2017)

Spatial transcriptomics normalized to normal tissue showed that IMCAFs colocalized with lymphocytes, whiles MRCAFs colocalized with epithelial cells (Foster et al., 2022). IMCAF/lymphocyte interactions were more frequent in later tumors. An *Acta2*-driven fluorescent reporter mouse showed that *Acta2* positive cells were very heterogeneous. This Visium analysis suggested that MRCAFS may differentiate into SSL/IMCAFs.

Cross species comparison with human breast cancer samples (n = 3 patients with variable clinical features) revealed consistent CAF classification and gene expression. Three human MRCAF clusters also associated with focal adhesion, MGP, and FOSB expression. *DPP4+* SSLCAFs displayed high *TIMP2* levels. *PDGFRb* and *POSTN* expression were in fibrotic fibroblast clusters. FAK depletion *in vivo* increased in SSLCAF presence and tumor growth, while depleting 2 CAF populations, emphasizing MRCAF importance in tumor progression (Foster et al., 2022).

Analysis of mesenchymal cells from *MMTV-PyMT* mouse tumors revealed 3 spatially separated CAF subpopulations, including peri-vascular (vCAF), mammary fat pad (myCAF), and transformed epithelium (dCAF) (Bartoschek et al., 2018). Surprisingly, immunomodulatory functions were not significant in any of the CAF populations. VCAFs expressed vascular regulators such as *Notch3*, *Epas1*, *Col18a1*, *Nr2f2* and were located in the tumor core near vasculature, while myCAFs expressed many ECM genes, such as glycoproteins (*Dcn*, *Lum*, and *Vcan*), *Col14a1*, *Fbln1*, *Fbln2*, *Smoc*, *Lox*, *Loxl1*, and *Cxcl14*. DCAFs had strong expression of stem cell genes (*Scrg1*, *Sox9*, *Sox10*, *CD10*, *Gpr77*), were near the tumor-stroma boundary, and could be derived from post-EMT tumor cells. The myCAF proportion decreased with tumor progression while vCAFs increased (Bartoschek et al., 2018). This classification scheme was very distinct from the Foster classification scheme, in spite of using the same murine model.

Another spatial transcriptomics study identified CAF populations from eight human breast cancer patients (Mao et al., 2023). Normal fibroblasts displayed high *CCL2*, *TNFAIP6*, *IRF1*, and *TAC1* expression (Mao et al., 2023). CAFs were high in collagens, *FN1, SPARC,* and *CTHRC1*; genes with low expression in CAFs correlated with a favorable prognosis. Spatial transcriptomics revealed these cells were spread throughout the tumor. Wound healing CAFs were high in *CCN5, TNXB, IGFBP5* and *IGFBP6*, and associated with an immunosuppressive environment, and stress responsive CAFs were enriched for heat shock protein and *AP1* (Mao et al., 2023).

### 3.5 Breast CAFs with immunomodulatory capabilities

Studies in immunocompetent mouse models have demonstrated that *Fap* expressing CAFs associated with an immune supportive environment (Costa et al., 2018). SCRNA seq analysis of 16 breast tumors (biased toward luminal breast cancer) with immunohistochemical follow-up revealed four distinct CAF subpopulations (Costa et al., 2018). Two myofibroblast subsets, including one with high ECM and inflammatory signatures (CAF-S1), and another cluster with a perivascular signature, had a stronger presence in triple negative breast cancer (TNBC) compared to other subtypes. CAF-S1 were close to tumor cells and correlated with CD45^+^ infiltration. CAF-S1 fibroblasts (*CD29* ^*Med*^ *FAP* ^*Hi*^ *FSP1* ^*Low-Hi*^ *aSMA* ^*Hi*^ *PDGFRb* ^*Med-Hi*^ *CAV1* ^*Low*^) promote an immunosuppressive environment by secreting CXCL12, and also attract and retain CD4^+^ CD25^+^ T lymphocytes with high expression of *OX40L*, *PD-L2*, and *JAM2* association. Interestingly, CAF-S1 increases T lymphocyte survival and promotes their differentiation into CD25^High^FOXP3^High^, through B7H3, CD73, and DPP4. This work was further confirmed by *in vitro* co-culture studies. Thus, inhibition of B7H3 or CD73 could target CAF-S1 cells to inhibit immunosuppression. Both CAF-S1 and CAF-S4 promote metastasis and immunotherapy resistance correlated with high FAP expression. FACS analysis for the classical markers (integrin B1/CD29, FAP, FSP, aSMA, PDGFRb, and CAV1) showed that removal of one of these markers changed the classification scheme, underscoring that these markers are not redundant and contribute different functions to different CAF subtypes. This study prompted for an interest in understanding how FAP expressing CAFs may mediate immune evasion.

Analyzing eight primary human breast tumors identified 8 *FAP+* fibroblast populations, following up on previous data emphasizing the importance of FAP (Kieffer et al., 2020). These populations included ECM-myofibroblast CAFs (myCAFs), detox-immunomodulatory CAFs (iCAFs), *IL*-iCAFs, *TGFβ*-myCAFs, wound-myCAFs, *IFNγ-*iCAF, IFNαβmyCAF, acto-myCAF (Kieffer et al., 2020). ECM-myCAF and TGFβ-myCAF, characterized by extracellular matrix proteins and TGFβ signaling respectively, indicated primary immunotherapy resistance. Populations were confirmed by FACS. ECM-myCAF upregulates PD-1 and CTLA4 protein levels in regulatory *FOXP3* ^*+*^ *T* lymphocytes, which subsequently increases *TGFβ*-myCAF content. This work suggests a positive feedback loop between CAF *FAP* ^*Hi*^ clusters and *FOXP3* ^*+*^ *CD4* ^*+*^ *CD25* ^*+*^Tregs contributing to immunotherapy resistance. Analysis of fibroblast populations and breast cancer subtypes showed broad enrichment for iCAFs in TNBC whereas luminal breast cancer showed myCAF enrichment. Other studies have shown that *COL1A1+* CAF localization near breast cancer cells was associated with poor T cell infiltration (Huang et al., 2023), while other analysis found high CAF *FGFR* was associated with cytotoxic T cell exclusion (Wu et al., 2022). Follow up of these studies with *in vivo* inhibitors may offer insight into novel therapeutic strategies to enhance checkpoint blockade therapy, leveraging the accessibility of paracrine molecular signaling.

### 3.6 SCRNA sequencing of CAFs and breast cancer patient outcome

Some scRNA seq data associate CAF subtypes/gene signatures with treatment resistance. Analysis of 70 human responder and non-responder samples showed that HER2 therapy resistance was correlated with *PDPN+* CAF enrichment, which showed enrichment for tryptophan signaling, worse prognosis, and suppressed NK cell activity (Du et al., 2023). Two different studies used scRNA seq to identify markers of endocrine therapy resistance; in the *MMTV-PyMT* model, *Cd63* expression was correlated with resistance (Gao et al., 2020), while human patient sample analysis showed that loss of *CD146* was associated with resistance (Brechbuhl et al., 2017). Data from human luminal breast cancer samples showed the high vCAF and myCAF signatures were associated with radiation resistance, tumor cell heterogeneity, and cancer stemness, while iCAF signatures and *CRABP5* and *CD53* expression were correlated with treatment response (Nandi et al., 2022). These studies all raise interesting questions regarding potential prognostic use of CAF gene signatures, however, functional follow up examining how cancer cells are impacted or whether intracellular signaling could be targeted would be useful first steps. See Table 3 for a summary of patient outcomes and CAF features in breast and ovarian cancer.

**TABLE 3:** Gene expression associated with favorable and poor prognosis in breast and ovarian cancer.

	Favorable prognosis/Survival	Poorer prognosis/Survival	References
Breast Cancer CAF Markers	Genes with low expression in myCAFs *RPS9, RPS8, RPS6, RPL38, RPL34*	*FN1, SPARC, CTHRC1,* *Col4a1, Tspan11, St8sia2, Tnfaip6, PDPN, POSTN, EDIL, GRP, IL16, PTN, TAC1* Higher CAF content Higher vCAF content	Mao et al. (2023), Huang et al. (2023), Sun et al. (2021), Nandi et al. (2022)
Ovarian Cancer CAF Markers	none identified	*TGF-β,* *NOTCH, SNA1L2, TGFBR1, WNT11, POSTN, ASMA, VIM, and PDGFRb* Higher CAF content	Olbrecht et al. (2021)

## 4 Single-cell sequencing of ovarian CAFs

High grade serous ovarian cancer (HGSOC) is the most common and lethal gynecological disease. It originates from the ovary, fallopian tube, or peritoneum (Kim et al., 2018). Frequently, ovarian cancer has already metastasized at diagnosis, and thus the 5-year survival rate is 30% (Siegel et al., 2017). CAFs are a critical component of the ovarian cancer TME, and CAF marker expression has been correlated with altered clinical outcomes. FAP (gene and protein) and PDGFRb expression has been correlated with poor ovarian cancer clinical outcomes (Mhawech-Fauceglia et al., 2015; Avril et al., 2017; Li et al., 2020) and chemotherapy resistance (Mhawech-Fauceglia et al., 2015; Avril et al., 2017; Zhang et al., 2022). Prior to scRNA sequencing in ovarian cancer, some data addressing CAF communication with other cell populations had been observed through co-culture studies and computational analysis. CAF heterogeneity was appreciated using tools such as microarray, but not fleshed out to the extent or with the interest level present more recently (Yeung et al., 2016).

### 4.1 Ovarian CAF SCRNA sequencing data and metastasis

SCRNA sequencing of 5 human primary and 3 metastatic HGSOC samples, 2 endometrioid carcinomas and 1 normal sample revealed five CAF populations (Deng et al., 2022). One cluster was *STAR* positive identifying normal ovarian stromal cells (Deng et al., 2022). The cluster most aligned with myCAFs displayed canonical marker expression, including collagen *COL1A1, COL3A1, CTHRC1*, and *FAP*, and was associated with metastasis. An immunomodulatory CAF cluster expressed high *CFD, OGN*, tumor suppressor gene *CCDC80*, and *PLA2G2A* levels (Deng et al., 2022). Another CAF cluster was enriched for growth factors *EGFR, IER2M,* and *KLF2*. The final cluster was characterized by nuclear-enriched lncRNAs, *MALAT* and *NEAT1* (Deng et al., 2022). Metastasis-derived CAFs were enriched for angiogenesis, EMT, and coagulation genes (Deng et al., 2022)*.* Hallmark pathway analysis confirmed that *CTHRC1* and *FAP* myCAF cluster was most associated with pro-tumorigenic angiogenesis, EMT, hypoxia, and PI3K/AKT/mTOR signaling (Deng et al., 2022); and the myCAF (*FAP, COL1A1,COL1A3*) cluster may be critical for tumor growth and metastasis. SCENIC analysis showed SOX2 and SRF transcription in myCAFs, while NFkb and STAT signaling were upregulated in the *MALAT/NEAT1* cluster. This clustering analysis was rather unique in identifying growth factor-expressing CAFs as a separate cluster, and utilizing SCENIC analysis to investigate regulation of these genes. Comparison with a normal sample was also commendable. See Table 4 for an overview of ovarian CAF populations and gene expression, as well as Figure 2.

**TABLE 4:** Ovarian CAF populations, ATAC, and scRNA seq data of genes associated with CAFs in ovarian cancer along with genes associated with short-term survival.

Functional classification	Single-cell ATAC-seq markers	Single-cell RNA-Seq markers	References
MyCAF	None reported	*COL1A1, COL3A1, CTHRC1,* *COL1A1,* *FAP* *VIM POSTN, ACTA2, FN1, MMP11*	Deng et al. (2022), Hornburg et al. (2021)
iCAF	None reported	*EGFR, IER2M, KLF2,* *C1QA/B/C, CFB, CXCL1,2,10,12, IL6, IL10*	Deng et al. (2022), Izar et al. (2020)
ImCAF	None reported	*CFD, OGN* *,* *CCDC80, PLA2G2A* *FAP* *, CXCL14, CCL2, Il1*	Deng et al. (2022), Hornburg et al. (2021)
Short-Term Survival	None reported	*POSTN* *CD* *36,* *aSMA, VIM, PDGFRb*	Ferri-Borgogno et al. (2023)

Recent long-read scRNA seq analysis of 3 patient samples with omental metastasis showed a connection by uMAP between myCAF and mesothelial cells only in metastatic samples (Dondi et al., 2023). Further analysis of 3′UTRs indicated that *COL1A2, COL3A1, COL5A2*, and *COL6A1* all displayed evidence of miRNA silencing, which has been associated with Mir29. Together with data from the literature, this work suggested a TGFb/miR-29/Collagen signaling axis associated with CAF differentiation from mesothelial cells (Dondi et al., 2023). This work was novel in using full-length UTR data to explore an established signaling axis in CAF differentiation, which could be further supported by future experimentation in mice and/or velocity analysis.

### 4.2 Ovarian CAFs and tumor immunity

Given the interest in optimizing checkpoint blockade therapy, various scRNA seq data connect CAF expression with lymphocyte presence and signaling. SCRNA seq of 15 patient derived ovarian tumors identified 3 fibroblast populations contributing to immune evasion: *IL1*CAFs, *TGFb*CAFs (myCAFs), and a third CAF cluster enriched for epithelial genes such as *KRT8, KRT15,* and *KRT18* (Hornburg et al., 2021). This third CAF cluster is not frequently found in the literature and bears further investigation. *IL1*CAFs, displaying *CXCL14* and *CCL2* expression, were associated with CD8^+^ T cell infiltration and CXCR4 activation. Here, *TGFB* myCAFs expressed reactive stroma markers *POSTN, ACTA2, FN1,* and *MMP11* and are enriched in tumors with low CD8^+^ T cell infiltration, suggesting the dense ECM hinders immune infiltration consisent with other studies (Hornburg et al., 2021). This work relied on fibroblast categorization by *COL1A1* and *PDGFRa* after flow sorting by EPCAM-CD45-exclusions, and may not have captured all CAFs. However, this is balanced by the CAF samples being run separately. These findings are very consistent with the broader literature connecting CAFs and immune infiltration.

SCRNA seq of 22 ascites specimens from 11 HGSOC patients identified two immunoreactive iCAF subpopulations (Izar et al., 2020). One cluster had high TNF-alpha/NF-KB target gene expression, while the iCAF2 cluster displayed high oxidative phosphorylation gene expression. There was significant interpatient variability in ascites cell gene expression, including immunoreactive CAF subpopulations expressing complement factors (*C1QA/B/C*, *CFB*), chemokines (*CXCL1,2,10,12*) and cytokines (*IL6, IL10*) (Izar et al., 2020). The iCAFs may be influenced by the M1/M2 macrophage populations in the ascites samples (Izar et al., 2020). These data suggest that immune cell infiltration is associated with omental metastasis, although the causal intracellular interactions are not explored (Olalekan et al., 2021). This work also revealed that CAF classification by TCGA subtypes (differentiated, proliferative, mesenchymal, and immunoreactive) showed that all six malignant clusters expressed “differentiated” and “proliferative” signatures, whereas the “mesenchymal” and “immunoreactive” signatures were only present in the CAF and immune cells, as opposed to malignant cells (Izar et al., 2020). Overall, data underscore the relative importance of CAFs in immune infiltration, and the importance of TGFb in this process, opening the door for translationally relevant investigation of these intracellular interactions.

### 4.3 Spatial transcriptomics of ovarian CAFs

Spatial transcriptomic analysis compared two patients with short term survival (<2 years) and long-term survival (>10 years) (Ferri-Borgogno et al., 2023). In short term survival samples, 2 stroma clusters near the tumor edge expressed high *COL1A1* and *POSTN* consistent with myofibroblast phenotype, while two stroma clusters distal to the tumor edge expressed high *COL1A1* and *CD36* (peri-vascular). High *POSTN* and *CD36* expression was not associated with long-term survival, but was present with more aggressive disease. Here, only two of six stromal clusters in the long term survival samples expressed high expression of the canonical CAF markers *POSTN, ACTA2, VIM*, and *PDGFRb*; while all short term survival clusters expressed these CAF markers; suggesting the association of these markers with tumor progression and worse prognosis. Short term survival samples are enriched for *SMA,VIM,* and *PDGFR* CAFs, and show more crosstalk between CAF APOE and tumor LRP5 at the tumor-stroma interface, supported by staining and *in vitro* experiments (Ferri-Borgogno et al., 2023). Upregulation of these signaling pathways increase tumor cell proliferation and survival (Ferri-Borgogno et al., 2023). While this study had a small n, it leverages spatial transcriptomics and ligand receptor analysis to highlight potential therapeutic and prognostic relevance of CAF markers and localization, supported by *in vitro* findings as well.

### 4.4 Ovarian CAF gene expression and intracellular interactions

SCRNA seq has also been leveraged to investigate CAF-stroma crosstalk in tumorigenesis. Combining public and new scRNA-seq data, a recent study profiled the TME of 10 solid cancers including ovarian (HGSOC) addressed plasticity and stromal cell interactions (Luo et al., 2022). After classifying clusters by markers (including *DCN* and *COL1A1* for fibroblasts, lymphocytes by *CD3D* and *CD3E*, and *CD68* and *CD14* for myeloid cells)*,* Interaction analysis showed highest crosstalk among fibroblasts, endothelial cells, and myeloid cells. Fibroblasts had the most interactions with other TME cells across cancer, with ovarian cancer showing some of the strongest fibroblast-epithelial crosstalk (Luo et al., 2022; Chai et al., 2024). These data emphasize the importance of CAFs particularly in ovarian cancer (Luo et al., 2022). These data, indeed most scRNA patient data, focus on HGSOC/advanced disease and may be limited by clustering according to two CAF markers initially; future investigations may evaluate these findings in earlier stage ovarian cancer. This work does raise interesting hypotheses bearing further functional investigation, and fit with other ovarian cancer data showing canonical CAF markers/myCAF association with advanced disease.

A different study analyzed bulk RNA seq of eight ascites samples from serous ovarian cancer, tumor-derived organoids, CAFs-enriched (eCAFs), and malignant effusion with eight normal ovarian tissues together with scRNA seq of 4 ascites samples (Carvalho et al., 2022). Analyzing CAF derived-ligands and epithelial cancer cell receptor expression showed enrichment for PI3K-AKT, focal adhesion, and EMT signaling (Carvalho et al., 2022); suggesting a putative synergistic benefit for PI3K inhibition as a targeted therapy in ovarian cancer (Luyuo Guan, 2018). Mapping the most relevant genes associated with PI3K-AKT revealed that myCAF- derived collagens, fibronectin, vitronectin, laminin, and osteopontin displayed strong interaction with integrins (e.g., *ITGB5*) in cancer cells. Collagens, *MIF, MDK, APP,* and laminin were the most highly expressed, and the top ligand-receptor interactions were CAF derived *THBS2/THBS3* with cancer cell *CD47*, and CAF-derived *MDK* with cancer cell *NCL/SDC2/SDC4*. Interestingly, two subpopulations of CAFs were highest for incoming signaling. While these data include a mix of bulk and scRNA seq, the use of normal ovarian tissues is commendable, and supports the potential utility of experiments on signaling interactions in mouse and co-culture study.

### 4.5 SCRNA sequencing of ovarian CAFs and patient outcome

**FIGURE 3:**
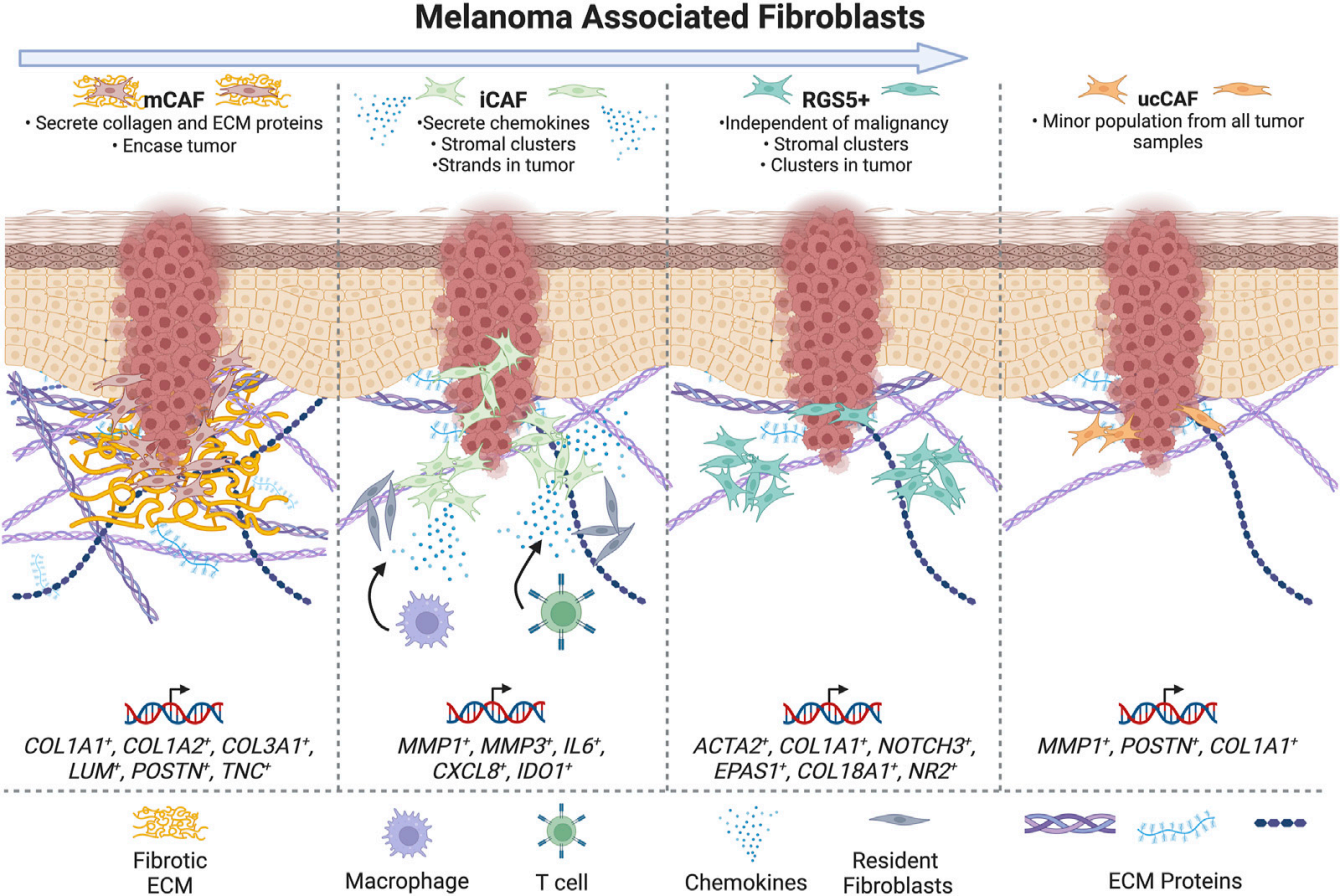
Schematic summarizing CAFs populations in melanoma -- summarizing subtypes in categories with highest concordance and markers of treatment resistance and survival. The arrow at the top of the figure indicates increasing tumor progression moving from left to right.

ScRNA seq of HGSOC has yielded further associations with patient outcome (Zhao et al., 2022). A study of 7 treatment naïve patient HGSOC and 5 age-matched normal samples found that CAFs expressing 6 genes were associated with worse prognosis and immunotherapy response relative to myCAFs (Zhao et al., 2022). The proportions of these populations varied greatly between patients. Tumor promoting CAFs displayed high expression of *CCDC80, SFRP2, VCAN, COL8A1, RGS5, HIIGD1B, NOTCH3, and NDUFA4L2* (Zhao et al., 2022). Different analysis of these TCGA data predicted worse patient outcome with high *NOTCH, SNA1I2, TGFBR1,* and *WNT11* expression (Xu et al., 2022). The *ACTA2, VIM, COL3A, COL10A,* and *MMP11* myCAFs were the dominant CAFs in HGSOC tumors and could induce cancer cell EMT *in vitro* (Xu et al., 2022). Another scRNA seq analysis of 5 HGSOC ascites samples identified 2 myCAF subpopulations (Li et al., 2022). Both CAF populations expressed high *VIM* and *COL1A1*, while *ACTA2* was high in only one population and was co-expressed with *SNAI1, ZEB1,* and *TWIST1* EMT transcription factors, and angiogenesis genes (Li et al., 2022). The other CAF subtype showed enrichment for the apical and basal part of the cell, together with cell substrate junctions (Li et al., 2022). SCRNA seq of 18,403 cells from 7 treatment-naïve ovarian cancers identified 32 stromal cell populations, with myCAF *TGFb* correlated with poor patient outcome and a 10 gene predictive signature being prognostically useful as well (Olbrecht et al., 2021). These finding may warrant further validation given the use of tSNE versus uMAP. Overall, dominant myCAF and ECM gene expression associate with advanced disease. Future studies may address targeting the intracellular signaling originating from myCAFs, with velocity and functional studies further enhancing our understanding of when such an intervention may be most beneficial. This work also highlights the relevance of CAF EMT. Refer to Table 3 and Figure 3 for a summary of data regarding patient outcomes and CAF features.

## 5 Single-cell sequencing of melanoma CAFs

While this review focuses on breast and ovarian CAFs, it is also logical to directly compare wound healing fibroblasts and melanoma/skin CAF scRNA seq data. CAF function in melanoma versus breast and ovarian cancer may be quite different; while melanoma, breast cancer, and ovarian cancer are all solid tumors, breast and ovarian cancers are carcinomas, while melanoma is not. All three cancers have distinct causes of patient mortality and metastatic sites. While melanoma generally displays high CD8^+^ T cell infiltration and responds to immune checkpoint blockade therapy, breast and ovarian tumors generally display less CD8^+^ T cell infiltration and poorer immunotherapy response. Canonical CAF markers also have different functions in these cancers; for example, aSMA can be expressed by basal cells in the breast, while vimentin is frequently expressed by melanoma cells and is associated with poor prognosis (Mumcuoglu et al., 2010; Novotný et al., 2020).

Melanoma comprises up to thirty subtypes, with six major subtypes. Prior to scRNA sequencing, CAF heterogeneity data was largely focused on comparisons between normal dermal fibroblasts and CAFs early and later in tumor progression, and testing whether CAFs/fibroblasts were tumor promoting or inhibiting. Overall, it is thought that normal dermal fibroblasts initially inhibit tumorigenesis via signaling to tumor and immune cells and generating a tumor-limiting ECM, but with intercellular interactions and time, these CAFs become tumor promoting, secrete growth factors, upregulate FAP and NOTCH1, and bind N cadherin (Zhou et al., 2015; LeBleu and Kalluri, 2018). Some data correlate CAF markers/presence and patient prognosis in melanoma. Of note, VIM and aSMA identify melanoma cells and normal fibroblasts respectively and are not unique CAF markers in melanoma (Novotný et al., 2020). Immunostaining of 117 human melanomas showed FAP expression negatively correlating with progression free survival in the absence of immunotherapy (Wong et al., 2019). Low FSP/S100A4 expression has been correlated with improved disease free survival (Andersen et al., 2004), while data showed high nuclear S100A4 expression correlated with worse overall survival (Jurmeister et al., 2019); however, these studies do not differentiate between CAF and tumor S100A4 expression. TGFb, presumably CAF derived, is also associated with poorer patient prognosis (Kodama et al., 2021).

### 5.1 SCRNA sequencing of mouse models of melanoma/skin cancer

Relative to breast and ovarian cancers, melanoma and skin cancers have fewer scRNA sequencing data. One study analyzed tumors from B16F10 tumor cells in mice with host eGFP expression to verify stromal identity (Davidson et al., 2020). Tumor and lymph node cells were subjected to scRNA sequencing, producing 3 stromal clusters (Davidson et al., 2020). The S1 iCAF cluster displayed high *Pdpn, Pdgfra*, *Cd34,* and *Cxcl12, Csf1, IL6,* and complement factors *C2, C3,* and *C4B e*xpression (Davidson et al., 2020). Immunostaining confirmed high CXCL12 expression in this population (Davidson et al., 2020). The S2 cluster showed high *Pdpn* and *Pdgfra,* low *Acta2* and *Cd34*, and high expression of ECM-associated genes including *Postn* and *Tnc*; as a result, the authors conclude that this CAF population is associated with desmoplasia (Davidson et al., 2020). The S3 population displayed high *Acta2;* contractile gene expression including *Rock1, Mcl2,* and *Mlck;* and *Col1a1*, *Col1a2, Fn1,* and *Sparc*. Since the S3 cluster also expressed pericyte-associated genes such as *Ng2,* this population likely includes fibroblast-like cells that have separated from the vasculature. This conclusion was supported by immunostaining and NG2 and aSMA colocalization (Davidson et al., 2020). With respect to established CAF markers, scRNA seq and immunostaining showed that PDPN and PDGFRa largely colocalize, whereas aSMA+ cells were distinct (Davidson et al., 2020).

Analysis of the *mKi67* proliferation gene suggests that CAF proliferation increases later in tumorigenesis (Davidson et al., 2020). Flow cytometry to test for the presence of the 3 CAF populations in normal skin showed that the S1 and S2 populations were present in normal skin, while the S3 population was rare. This fit with the scRNA seq finding showing S1 and S2 enrichment in earlier stage tumors (Davidson et al., 2020). As expected, the S1-S3 populations engaged in different intracellular interactions. S1 CAFs were enriched for NK cell interactions via CXCR1 expression, and displayed gene expression associated with both T cell recruitment and suppression (Davidson et al., 2020). The S1 and S2 populations also displayed strong C3/CSF1 interactions with macrophages, and with NK cells via CXCR1. C3 inhibition *in vivo* slowed tumor growth, and altered macrophage and T cell infiltration. This study had many strengths including consistent *in vivo* immunostaining/validation, use of temporal follow up and intracellular interaction analysis, and data from normal human fibroblasts. There may be some limitations due to the use of highly aggressive B16F10 melanoma cells.

Previous data revealed that loss of *MacroH2a*, a histone modifying gene, elicited an expanded population of CAFs with canonical activation markers in *BRAF/pTEN* deficient mice, a smaller myofibroblast-like CAF population, and larger tumors (Filipescu et al., 2023). CAF clustering did not yield the typical iCAF/myCAF/vCAF/apCAF clusters; rather, 4 clusters were defined by high gene expression as follows: *Fap+Pdgfra+Meg3+*; *Wif1+* (also expressed in papillary dermis); *Acta2+Lrrc15+; Acta2+ Fbln1+;* and *Zeb2* (Filipescu et al., 2023). Consistent with previous literature on CAF markers, this analysis found that *Fap, Acta2, and Pdgfra/b* were not uniformly expressed across the CAF clusters, and expression of these genes did not all correlate with each other (Filipescu et al., 2023). SCRNA sequencing of *MacroH2a -*deficient murine tumors showed increased strength of CAF interactions with a subset of tumor cells (Filipescu et al., 2023). This work represents an excellent source of gene expression profiles for different CAF populations using a more physiological mouse model of melanoma, and leverages different RNA seq associated tools such as spatial transcriptomics and ligand receptor interactions, although the focus of the paper was rather broad. This study also highlights *Meg3* as a physiologically relevant CAF gene warranting further experiments to define its role in melanoma CAFs.

### 5.2 SCRNA sequencing of human patient melanoma and skin cancer

An *in vitro* analysis provided some useful context for understanding CAF heterogeneity and gene expression in spheroid culture. SCRNA sequencing of G361 melanoma cells in spheroid co-culture with less-damaged juvenile (n = 1) or adult UV damaged fibroblasts (n = 1) samples identified 3 fibroblast subpopulations across both types of samples (Novotný et al., 2020). The ECM-fibroblast cluster displays high chemokine expression including *CXCL8* and low ECM-associated gene expression. The ECM-cluster showed enrichment for cytokine, Toll-like, and Nod-like receptor KEGG signatures. A second CAF cluster with high *ID* gene expression associated with cell fate, displayed focal adhesion and TGFb KEGG signature enrichment; while the ECM+ cluster displayed highest expression of genes such as *COL1A1* (Novotný et al., 2020). Relative to juvenile fibroblasts, adult normal fibroblasts had broader clusters, higher *LRRC15* and *CTHRC1* and inflammation-associated gene expression, and downregulation of *MGP* (Novotný et al., 2020). Both melanoma cells and fibroblasts expressed *VIM* as expected (Novotný et al., 2020). This study mostly examines how melanoma cells can modify normal fibroblast gene expression and the reliance of these changes on fibroblast age/damage status, rather than examining CAF heterogeneity. While this study offers insight into gene expression in spheroid culture, one limitation regarding the broad applicability of these gene signatures includes that fibroblast clustering was based on solely *FAP* expression, adding bias and limiting CAF heterogeneity. It would be interesting to see if these gene signatures repeat in other melanoma cell lines in spheroid culture, and examine *ID* function in melanoma CAFs.

Another study broadly examined skin cancer CAFs by scRNA sequencing of >5,700 cells from 3 human melanoma, 4 squamous cell carcinoma (SCC), and 3 basal cell carcinoma (BCC) samples with adjacent normal skin (Agnes et al., 2023). Cells were categorized into 5 categories, which for fibroblasts included *COL1A1* and *PDGFRA* expression (Agnes et al., 2023). The following CAF clusters were observed: matrix, immune, *RGS5+*, and unidentified CAFs (uCAF) (Agnes et al., 2023). Matrix CAFs showed high expression of *COL1A1*, *COL1A2*, *COL3A1*, *LUM*, *POSTN*, and *TNC*, and largely consisted of SCC and BCC CAFs (Agnes et al., 2023). Immunomodulatory CAFs (iCAFs) displayed high expression of *MMP1, MMP3, IL6*, *CXCL8,* and *IDO1,* and included CAFs from melanomas, 1 SCC, and 1 BCC (Agnes et al., 2023). UCAFs were a minor population. These data were validated by mapping matrix and matrix remodeling genes to the matrix mCAF population, and mapping chemo- and cytokine genes to iCAFs (Agnes et al., 2023).

The *RGS5+* population displayed high *ACTA2* and *COL1A1* expression consistent with myofibroblast identity, and includes pericytes. Here, pericytes did not generate a separate cluster (Agnes et al., 2023). *RGS5+* cells also displayed high expression of previously published vCAF markers (Bartoschek et al., 2018)*,* but lacked expression of the *CDH5, PECAM1, TIE1* or *CD62* endothelial markers (Agnes et al., 2023). The lack of a separate pericyte cluster is consistent with other publications, and was also confirmed by a separate analysis of head and neck carcinomas (Agnes et al., 2023). The *RGS5+* population also shared markers with the vascular smooth muscle cell cluster (Agnes et al., 2023). *RGS5+* CAFs were most widely present in these tumors (Agnes et al., 2023). *RGS5* staining showed that these cells had both stromal and perivascular tumor localization, while in adjacent normal tissue, *RGS5+* cells were only near blood vessels. These data are fit with data from other cancers suggesting that pericytes can undergo a pericyte-fibroblast transition. This work highlights the challenges of dissecting pericyte versus fibroblast gene expression. Future studies could investigate PFT in melanoma using Pseudotime cell-fate mapping analysis. Additionally, while *RGS5* is frequently identified in CAF populations including in other cancers, *RGS5* CAF function is not well defined.

This work also offered some insights into established CAF markers. Here, *FAP* and *ACTA2* included all CAFs, as well as vSMCs (Agnes et al., 2023). *COL1A1* was an effective pan-fibroblast marker (Agnes et al., 2023). Unexpectedly, *PDGFRA* staining was weak in both tumor and adjacent normal tissues. *ACTA2* expression was strongly expressed in normal tissues and CAFs (Agnes et al., 2023).

Staining for mCAFs and iCAFs was done to localize these cells in 39 samples (Agnes et al., 2023). MCAFs were identified by *COL11A1* and *PTGDS* staining, while iCAFs were identified by *MMP1*, and *COL1A1* was used as a pan-fibroblast marker (Agnes et al., 2023). Staining revealed MCAF localization in large patches encasing tumor cells and at the tumor-stroma border, while iCAFs were intermixed with *MMP1-, COL1A1* cells clustered within tumors. *COL11A1* was strongly aligned at the tumor border and correlated with T cell exclusion, similar to other studies. See Table 5 for a summary of melanoma CAF populations and gene expression.

**TABLE 5:** Melanoma CAF populations, ATAC and scRNA seq data of genes associated with CAFs in melanoma along with genes associated with short-term survival and treatment resistance.

Functional classification	Single-cell ATAC-seq markers	Single-cell RNA-Seq markers	References
mCAF	None reported	*COL1A1, COL1A2, COL3A1,LUM, POSTN,TNC, COL11A1, PTGDS, Acta2, Rock1, Mcl2, Mick, Col1a1, Col1a2, Fn1, Sparc*	Agnes et al. (2023), Davidson et al. (2020)
iCAF	None reported	*Pdpn, Pdgfra, Cd34, Cxcl12, Csf1, Il6, C2, C3, C4b, Postn, Tnc, Ng2, Zeb2, Acta2, Lrrc15, Fbln1, MMP1, MMP3, IL6,CXCL8, IDO1, MMP1*	Davidson et al. (2020), Filipescu et al. (2023), Agnes et al. (2023)
Short-Term Survival or Treatment Resistance	*FAP, FSP, S100A4, TBF-b*	*CCN2*	Wong et al. (2019), Andersen et al. (2004), Kodama et al. (2021) Hutchenreuther et al. (2018)

This study also presented findings on CAFs and tumor immunity. Comparing CAFs and normal fibroblasts showed that only CAFs express high *HLA*, underscoring the specificity of CAFs in tumor immunity (Agnes et al., 2023). Although the tumor n was low, significant differences in the chemo- and cytokine profiles in different cancers were observed; melanomas displayed higher *CXCL1-8* and *IL1B*, while SCC and BCC expressed higher *CXCL9-13, TGFB3,* and *LGALGS9*. *CXCL12* expression was high across all tumors in this study (Agnes et al., 2023). Analysis of different melanoma data suggested that *CXCL2, 12,* and *14* were particularly high in melanoma. Additionally, these data suggest that iCAFs rather than tumor cells are the major source of chemokines and at least in melanoma, cytokines. Higher iCAF expression of cytokines versus tumor cells was observed in oral SCC. *In vitro* follow up showed that co-culture with metastatic melanoma cells or an SCC cell line induced chemo- and cytokine expression in normal dermal fibroblasts (Agnes et al., 2023).

Ligand-receptor analysis of the scRNA seq data indicates that mCAFs display high collagen and ECM gene expression, with the receptors are located on the immune cells and melanocytes (Agnes et al., 2023). ICAFs expressed many ligands binding T and NK cell receptors (Agnes et al., 2023). Overall, while this study was rather broad in utilizing cells from a few different types of skin cancer simultaneously, their inclusion of normal fibroblast samples and experimental follow-up using RNA scope and co-culture makes this work very important for the field.

A different study of >4,000 cells from 46 primary human melanomas and TCGA data showed that high tumor *CCN2* expression correlates with worse disease-free survival (Hutchenreuther et al., 2018). *CCN2-*dependent gene expression changes were largely in angiogenesis associated genes (Hutchenreuther et al., 2018). Mice with *Col1a1*-dependent *Ccn2* ablation bearing B16F10 tumors showed impaired expression of canonical CAF markers including *aSMA* with reduced tumor angiogenesis (Hutchenreuther et al., 2018). While this work does not include CAF clustering or analysis of canonical CAF markers, the identification of a non-canonical CAF marker associated with tumor progression and *in vivo* experimental follow up suggest that *CCN2* may be functionally important for melanoma CAFs, warranting further study and alignment with other CAF markers.

Another study used TCGA data also used by (Tirosh et al., 2016) to examine ligand-receptor interactions between S1 iCAF cells and macrophages in human melanoma (Davidson et al., 2020). Function blocking antibody inhibition of C3 in mice bearing established B16 melanoma tumors increased T cell recruitment, reduced macrophage infiltration, and increased LY6C+ recruitment, highlighting the importance of this key complement factor and providing a strong example of scRNA seq data providing an interesting initial finding as the basis for experimental follow-up to support the importance of these findings (Davidson et al., 2020).

### 5.3 SCRNA sequencing of melanoma/skin cancer and patient outcomes

Some data have identified correlations between CAF markers or populations and tumor progression/prognosis. In mouse B16F10 melanomas, S1 and S2 CAFs (iCAFs and desmoplastic CAFs) are more abundant earlier in tumor progression, while S3 myofibroblast/contractile CAFs are enriched in late tumor progression (Davidson et al., 2020).

SCRNA seq analysis of 4,600 cells from 19 melanomas revealed that CAF abundance correlated with high *AXL/MITF* expression in tumor cells (Tirosh et al., 2016). High tumor *AXL* expression was frequently present with high CAF *AXL* levels; and *AXL*-dependent gene signatures were different in melanoma cells versus CAFs (Tirosh et al., 2016). Here, CAFs were defined by *FAP, THY1, DCN, COL1A1, COL1A2, COL6A1, COL6A2,* and *COL6A3* expression (Tirosh et al., 2016). Tumor cell *AXL* expression was verified by immunostaining, but not in fibroblasts (Tirosh et al., 2016). While melanoma *AXL* is associated RAF/MEK inhibitor resistance, it is unknown whether CAF *AXL* is as well (Tirosh et al., 2016). A list of CAF genes positively associated with T cell infiltration, including *CXCL12, CCL19, PDL2*, and the *SERPING1, C1S, C1R, CFB,* and *C3* complement factors was identified (Tirosh et al., 2016). The presence of CAFs expressing these genes could correlate with checkpoint inhibition response, but has not been tested (Tirosh et al., 2016). C3 expression was verified by immunofluorescence of 308 tumor samples (Tirosh et al., 2016). *In vitro* experiments showed that complement expression decreased with culturing, underscoring the importance of using fresh fibroblasts for scRNA sequencing in this field (Tirosh et al., 2016). This work provides an excellent resource for CAF gene expression, including tumors of different stages including post-treatment matched samples. The role of AXL in fibroblasts would benefit from further functional follow-up or contextualization with other CAF markers/subtypes.

Matrix CAFs in SCC, BCC, and melanomas expressing high levels of *COL1A1, COL1A2, COL3A1, LUM, POSTN, TNC, COL11A1* and *PTGDS* were a larger constituent of benign tumors, while higher numbers of iCAFs expressing *MMP1, MMP3, IL6 CXCL8,* and *IDO1* and different chemokines were correlated with advanced disease (Agnes et al., 2023). This finding was further supported by *in situ* hybridization of unique tumors (Agnes et al., 2023). This work is important because it broadly addresses CAF gene signatures and populations in skin cancer prognosis, but is slightly limited by the use of different tumor types and a small n of each tumor (Agnes et al., 2023). This study lays the foundation for future work testing the potential for these CAF gene signatures as biomarkers for patient prognosis in specific tumor types (Agnes et al., 2023). While the analysis of CAFs in this study may have been slightly limited by categorizing CAFs by *COL1A1* and *PDGFRA*, this analysis also benefits from the inclusion of adjacent normal samples (Agnes et al., 2023).

A different scRNA seq analysis of 46 human melanomas found that tumors with high CAF *CCN2* expression was associated with worse disease free survival and more neoangiogenesis (Hutchenreuther et al., 2018). While *in vivo* follow up examining *Col1a1*-dependent *Ccn2* loss in mice support the angiogenesis result, the impacts on tumor progression were not discussed. This work offers an example of using scRNA seq as a tool to execute more hypothesis-driven research as opposed to broadly surveying heterogeneity.

Another scRNA seq study provided analysis of CAFs and immune status of 472 skin cutaneous melanomas, using a previously used dataset (Tirosh et al., 2016; Shi et al., 2023). Data showed fewer CAFs present with more cytotoxic T cells in female patient tumors- but without any significant different in progression free survival (Shi et al., 2023). CAFs enriched for TGFbeta signaling correlated with a suppressive immune environment. Also, there were significantly more CAFs in mutant BRAF melanomas versus NRAS melanomas; and increased CAF frequency was associated with more recurrence. Two categories of tumors, namely, immune cold-suppressive and immune cold-exhausted interestingly correlated with higher versus fewer CAFs, respectively, while both groups displayed overall worse survival. Thus, simply using the numbers of CAFs to predict for recurrence or survival in SC melanomas is not sufficient. While these correlative data inform our understanding of melanoma and skin cancer CAFs, unravelling how these CAF changes functionally impact not only tumor cells but also other cells of the tumor microenvironment such as endothelial cells will be insightful and instructive going forward.

## 6 Discussion

Our review of fibroblasts in wound healing, breast cancer, ovarian cancer, and melanoma highlights that scRNA sequencing has enhanced the depth of knowledge on the established themes of fibroblast plasticity and heterogeneity. These data underscore that all fibroblasts- and all activated fibroblasts-are certainly not equal. SCRNA seq data have also afforded opportunities to broadly compare expression and overlap of canonical CAF markers simultaneously. Employing unbiased algorithms has highlighted the importance of different fibroblast populations in patient outcomes, regulation of stromal constituents, and fibroblast heterogeneity in regenerative wound healing, breast and ovarian cancer, and in melanoma.

### 6.1 Wound healing fibroblasts

SCRNA sequencing data from tissue-injury wound healing studies have increased the resolution of fibroblast populations involved in this process. The use of well-defined mouse models and controls have allowed for conclusions informed by single-cell omics and leverage models with a more defined process relative to CAFs. In contrast to where up to 12 subclusters of distinct fibroblast subpopulations could be identified, wound healing fibroblasts generally displayed 2-4 clusters, including the mechanofibrotic, activated responder, remodeling and proliferator populations (Foster et al., 2021). This heterogeneity is more complex than normal dermal fibroblasts, thus far showing reticular fibroblasts and papillary fibroblasts clustering together and encompassing dermal fibroblast populations (Agnes et al., 2023). With respect to canonical fibroblast markers, normal fibroblast data generally confirm the specific alignment of established normal fibroblast markers with their expected populations, and support the use of *CD90* as a skin fibroblast marker (Agnes et al., 2023). The exception was *DPP4*, a putative papillary fibroblast marker expressed in both skin fibroblast populations, suggesting the utility of further experimentation or future reliance on other markers (Agnes et al., 2023). Data support the heterogeneity of canonical activation markers; *Pdgfra* remains an important relatively broad marker, but does not include *Acta2/aSMA+* fibroblasts. *Fsp* and *Fn1* have different functions and are associated with specific subsets of fibroblasts as well. Sequencing studies have shown *Col1a1* to be largely associated with mechanoresponsive, less differentiated fibroblast populations.

In addition to providing insights into canonical fibroblast marker heterogeneity, scRNA seq studies have identified less-studied genes that may be critical for wound healing fibroblast identity such as *Mest, Spp,* and *Runx1*, which warrant exploration of their functional roles in wound healing fibroblasts (Guerrero-Juarez et al., 2019; Phan et al., 2021; Foster et al., 2022). Ligand receptor analysis has largely emphasized interactions with macrophages.

In contrast to much of the data presented here, a strength of wound healing scRNA seq data includes the incorporation of parallel approaches, such as ATAC seq or spatial transcriptomics, and *in vivo* lineage tracing. The use of parallel approaches makes sense with the availability of established mouse models with defined time frames for healing, and reliable access to fresh samples appropriate for scRNA sequencing. In contrast, a temporal switch in melanoma CAFs from pro-to anti-tumorigenic is still yet to be tightly defined. While no single marker encompasses all fibroblast populations, lineage tracing has offered insights into fibroblast lineages and helped construct models of spatial migration in the course of wound healing. Existing datasets in wound healing and contrasting with normal fibroblast data, perhaps including adjacent normal data, may be useful for identifying new therapeutic targets or biomarkers of regenerative versus scar wound healing.

### 6.2 Breast CAFs

Relative to ovarian cancer, there are more breast cancer scRNA seq data in both murine and clinical samples. The field has revealed many interesting findings, including related to CAF heterogeneity. Some common CAF subtypes used in this literature include myofibroblast myCAFs, iCAFs, imCAFs, and vascular vCAFs. Other CAF subtypes identified include developmental CAFs (Elwakeel et al., 2019), perivascular like (Wu et al., 2020), and steady state like (Foster et al., 2022), which have not been identified in multiple studies. It may be constructive to subcategorize myCAFs going forward, potentially using wound myCAFs or a matrix CAF designation as reported in a breast cancer and melanoma study (Friedman et al., 2020; Agnes et al., 2023). It is promising that there was overall concordance in CAF populations between mouse and human studies in one analysis (Foster et al., 2022), suggesting that the use of mouse models to obtain samples earlier and to perform a wider range of interventions may be translatable.

Aside from fibrosis/ECM gene signatures, growth factor, stemness, and EMT signatures are frequently highly expressed by CAF subpopulations. These data are interesting considerations, especially since many growth factors that have been pharmacologically targeted in tumors may target CAFs as well (Wu et al., 2022) and may be of interest to assess in ongoing clinical trials. With respect to canonical CAF markers, one theme in breast cancer CAFs from scRNA seq data includes use of these markers to stratify populations, such as by PDPN versus FSP (Friedman et al., 2020) or *Fap/Fsp/Acta2/Pdgfrb* status (Costa et al., 2018). Different studies showed myCAFs enriched for many canonical markers including *COL1A1/2, ACTA2, FAP, PDPN* (Wu et al., 2022)*.* Finally, other work clustered *Acta2* and *Pdgfrb* together, separate from *Fsp+* cells, with *Pdpn* only in a restricted population. It is important to note that *Col1a1, Postn, Pdgfra,* and *Dcn* were also observed in normal mammary fibroblasts (Sebastian et al., 2020).

Data from scRNA seq on CAF interaction with other cells and fibroblast cell lineage data are still emerging. Ligand-receptor analyses consistently support the importance and strength of interactions with immunomodulatory subpopulation of CAFs. Data show that some CAF subpopulations are important for cytokine secretion (Sun et al., 2021), while there is also a consensus that ECM-secreting CAF subpopulations may inhibit immune surveillance mechanically (Costa et al., 2018; Kieffer et al., 2020; Huang et al., 2023). CAFs are also involved in antigen presentation (Friedman et al., 2020). Some studies have also identified paracrine interactions between CAFs and immune cells including T cells, or upstream regulators such as FGFR in CAFs that may be druggable (Wu et al., 2022). It will be exciting to see how these data inform future studies refining checkpoint blockade therapy.

Ligand-receptor analysis also adds to our understanding of CAF-tumor cell interactions. Studies have thus far shown that some CAFs, including those with CD29^Med^ FAP^Hi^ FSP1^Low−Hi^ aSMA^Hi^ PDGFRb^Med−Hi^ CAV1^Low^ markers, interact more with tumor cells to promote tumor progression, are associated with aggressive disease, or alter tumor cell heterogeneity/stemness properties (Sun et al., 2021; Nandi et al., 2022). It may be constructive to investigate these questions using spatial transcriptomics going forward. CAF lineage data in breast cancer are still nascent and conflict; while some reports show that immunomodulatory iCAFs give rise to myofibroblast myCAFs (Chung et al., 2021), others report the opposite (Foster et al., 2022), highlighting the need for additional study perhaps with parallel approaches to understand fibroblast lineages. However it is understandable that these data are more limited given the requirements for more extensive data and samples to perform velocity analysis.

### 6.3 Ovarian CAFs

Relative to breast cancer, fewer studies with scRNA sequencing data have been published in ovarian cancer. These studies have largely focused on heterogeneity and intercellular interactions. Subtypes of ovarian cancer CAFs include myCAFs, iCAFs, and imCAFs, and less commonly, apCAFs. Differentially regulated CAF genes included EMT genes, ECM genes, and immunomodulatory genes as well such as chemokines. Classical marker expression was often spread across subtypes of CAFs, with some conflicting data on classification of ASMA in either the myCAF (Deng et al., 2022) or imCAF categorization due to ECM hindering immune capabilities (Hornburg et al., 2021). SCRNA seq data in this field has not yet addressed CAF subpopulation and treatment resistance or patient prognosis extensively, although the existing data generally associate myCAFs and *TGFb* with poorer outcome, as well as tumor biology relevant to tumor inflammation. There was good concordance with the breast cancer literature, including regarding immune infiltration being altered by CAF ECM secretion and interactions with Cd8+ T cell populations. Together with the breast cancer data, it seems that in addition to canonical marker genes, the putative markers such as *Il8* that should be considered going forward. As in wound healing, RNA sequencing studies have also identified new CAF genes of interest, such as *APOE* (Ferri-Borgogno et al., 2023). CAF lineage analysis data are not very prominent in the ovarian CAF scRNA sequencing literature yet, but these studies will be interesting to compare with other cancers. These publications were overall strong, however, future studies will benefit from higher n’s, and the inclusion of samples less biased toward aggressive HGSOC or ascites samples, and pre- and post-chemotherapy matched samples to evaluate CAF evolution. Additionally, ovarian CAF studies may benefit from use of parallel -omics approaches, since CAF lineage data are nascent, and functional follow up.

### 6.4 Melanoma/skin CAFs

The data on melanoma CAFs are still emerging, especially relative to breast cancer, and existing data often reflect the push to address treatment resistance to checkpoint blockade therapy and drugs targeting melanoma specific mutants such as BRAF. Numerous correlations with prognosis or advanced disease have been identified and there is currently a lack of consensus; iCAFs have been correlated with advanced disease (Agnes et al., 2023), as well as myCAFs(Davidson et al., 2020). Other specific genes associated with altered prognosis include *CCN2, TGFb, AXL,* and *C3* (Tirosh et al., 2016; Hutchenreuther et al., 2018; Shi et al., 2023). Melanoma CAF subpopulations usually include iCAFs and myCAFs, however, a number of other unique clusters have been put forth including, distinct ECM and contractile CAFs in (Davidson et al., 2020), *Wif1* and *Zeb2* subpopulations (Filipescu et al., 2023), and matrix and *RGS5* (Agnes et al., 2023). Similar to other CAF data, scRNA seq of melanoma and skin cancer CAFs confirms that canonical markers often overlap, but not completely; while Pdpn and Pdgfra colocalize, aSMA/Acta2 may be a different population in melanoma. Consistent with the literature, the PDGFR and Col1a1 canonical CAF markers continue to be broadly very important in melanoma-however, this could be due to initial clustering based on *Col1a1* in many cases. Future studies may continue to elucidate relative importance of some CAF markers shown to be important in data reviewed here, including *RGS5, AXL, and CCN2,* and may address overlap with these canonical CAF markers. Ligand receptor analysis was not common in many of these studies, but data support consistent CAF interactions with NK and T cells.

Future scRNA seq analysis in melanoma would benefit from analysis of non skin cutaneous melanoma subtypes, since existing data are biased toward these tumors. Relative to other cancers such as ovarian scRNA seq data from melanoma CAFs have advanced the field of understanding CAF- immune interactions considerably consistent with interest in overcoming checkpoint blockade therapy resistance in melanomas. Melanoma and skin cancer- and breast cancer- CAF data have begun distinguishing apCAF and iCAF populations. It remains to be seen whether these two separate populations are consistently present in other tumors, or display consistent gene expression. As melanoma scRNA seq data emerge, these studies may benefit from further inclusion of mouse work to obtain larger datasets suitable for velocity analysis, robust validation such as FISH, or for parallel analysis such as ATAC seq or mass spectrometry.

### 6.5 Fibroblast activation in wound healing versus cancer

The comparison between melanoma and skin cancer CAFs and wound healing fibroblasts is the most direct. These reports show that CAFs and wound healing fibroblasts express many common ECM components including *Col1a1, Col1a2, Col3a1, Fn1,* and *Sparc*; however, CAFs may express higher *Tnc, Coll11a1*, and *Col6a1/2/3* relative to wound healing fibroblasts. Data support the physiological importance of COL11A1 specifically in cancer. EMT genes were highly expressed in both wound healing fibroblasts and CAFs, and FAK dependent signaling was shown to play a similar role. Additionally, Wnt and TGFb signaling are important in both wound healing fibroblasts and CAFs. Wound healing fibroblast clusters included populations displaying high *Spp1, En1, Mest,* and *Crabp1* expression, which were not consistently observed in skin cancer CAFs; on the other hand, skin cancer CAFs showed fibroblast populations expressing high levels of complement, *Meg3, Lrrc15,* and *Rgs5,* which were not reported in wound healing CAFs. Further study of these differentially regulated genes in wound healing and CAFs, and understanding what genes are downregulated in CAFs relative to normal/wound healing fibroblasts would be informative.

Intracellular interaction analysis in wound healing fibroblasts, especially regarding immune interactions, has largely focused on macrophages. Data on ligand-receptor interactions in melanoma are still sparse, but emphasize interactions with T and NK cells. Further analysis is needed to compare how wound healing fibroblasts versus skin cancer CAFs interact with macrophages and T cells. Future studies may also compare skin cancer CAFs with wound healing CAFs as well as quiescent dermal CAFs which have relatively few data, to provide more understanding of activation versus quiescence. Another gap in knowledge is the different functions of fibroblast antigen presentation in CAFs and wound healing.

Comparing wound healing fibroblasts and other CAF populations, overall, scRNA sequencing data show that highly expressed CAF genes can display similarities with wound healing fibroblast signatures, including less studied genes such as *CRABP1, SPARC* or *RGS5*. Wound healing and CAF studies both emphasize that CAFs react to (as well as modulate) the mechanical environment since wound healing and CAFs show signatures associated with FAK (Foster et al., 2021; Foster et al., 2022). Both wound healing and CAF studies show with greater resolution that all previous marker genes associated with activation are not all equal, and are often not co-expressed. This finding fits with the discordant data regarding canonical markers and prognosis in breast cancer. One significant area of divergence between wound healing fibroblasts and CAFs is the interactions with immune cell populations. While wound healing fibroblasts interact with immune cells such as macrophages, CAF scRNA seq data emphasize interactions with innate immune lineages and adaptive immune cells. It is unknown to what extent cytokine/chemokine signaling is modulating tumor cells as well, since these receptors can be expressed.

Meta-analyses in the CAF and normal wound healing fibroblast fields may yield consensus terminology, reflecting greater heterogeneity than the common iCAF and myCAF designation. Consensus nomenclature and gene signatures will also reduce the need for individual marker protein-based analysis or lineage tracing, which likely bias data. Meta-analysis may help resolve issues such as two different studies using *MMTV-PyMT* and achieving divergent results (Bartoschek et al., 2018; Foster et al., 2022). Future studies would benefit from increasing clinical sample n’s, including normal fibroblasts, and includings pre- and post-treatment samples to identify potential therapeutic targets. Additional challenges for the CAF field include separating CAFs from vSMCs and pericytes; indeed, staining or spatial analysis may be necessary to separate these studies.

SCRNA seq data from cancer continue to reinforce the importance of canonical CAF markers, including with respect to clinical outcome, and have also uncovered novel areas for future study, including CAF lineages, and how EMT and stemness genes contribute to CAF function.

## Abbreviations

αSMA, alpha smooth muscle actin; apCAF, antigen presenting CAF; CAF, cancer associated fibroblast; ECM, extracellular matrix; EN-1, engrailed 1; FAP, fibroblast activation protein; FAK, focal adhesion kinase; FSP, Fibroblast specific protein; HGSOC, high grade serous ovarian cancer; iCAF, inflammatory CAF; myoCAF, myofibroblast CAF; PDGFR, platelet derived growth factor; PDPN, podoplanin; POSTN, periostin; scRNA seq, single cell RNA sequencing; TGFbeta, transforming growth factor beta; TME, tumor microenvironment; TNBC, triple negative breast cancer; VIM, vimentin.
